# Ethical Considerations and Recommendations for Humanizing Immigrant Language in Health Equity Data Collection, Reporting, and Measurement

**DOI:** 10.1089/heq.2024.0127

**Published:** 2025-05-27

**Authors:** Andrea Thoumi, Olurotimi Kukoyi, Kamaria Kaalund, Yazmin Garcia Rico, Rosa M. Gonzalez-Guarda, Jay Pearson, Viviana Martinez-Bianchi

**Affiliations:** ^1^ Department of Population Health Sciences, Duke University School of Medicine, Durham, North Carolina, USA.; ^2^ UNC-Chapel Hill, Chapel Hill, North Carolina, USA.; ^3^ Johns Hopkins Bloomberg School of Public Health, Baltimore, Maryland, USA.; ^4^ Independent Researcher, Greensboro, North Carolina, USA.; ^5^ School of Nursing, Duke University, Durham, North Carolina, USA.; ^6^ Duke University Sanford School of Public Policy, Durham, North Carolina, USA.; ^7^ Family Medicine & Community Health, Duke University, Durham, North Carolina, USA.

**Keywords:** data equity, health disparities, health equity, immigrant health, Latinx health, population health

## Abstract

Collecting accurate and consistent sociodemographic data is needed to improve health measurement and public health interventions. Missing or inaccurate data hinders the adequate assessment of the state of access, quality, and coverage in the overall population and communities experiencing social marginalization. Health measurement requires data labels that humanize all populations living, working, and residing across the United States and territories. Humanization is fundamentally grounded in the concepts of human dignity and ethical identity integrity. An often-overlooked form of exclusion in health care is the long-standing use of dehumanizing language, including its use in health measurement and data collection efforts, to refer to immigrant populations. In this perspective, we delineate ethical concerns regarding the use of dehumanizing language when referring to immigrant populations. We provide recommendations for health providers, researchers, and policy makers in improving humanizing language in health equity data collection and reporting through engagement of community experts, use of alternative language, implementation, and monitoring.

## Introduction

Collecting accurate and consistent data on race and ethnicity, preferred language, and sexual orientation and gender identity has the potential to improve health measurement and interventions, while closing gaps in access, quality, and coverage among historically marginalized and minoritized populations.^1^ For example, during the COVID-19 pandemic, policy makers increasingly used population-level disadvantage tools, such as the Social Vulnerability Index, to inform public health interventions. These tools use race, ethnicity, and other indicators of social distinction to identify communities and populations needing additional resources to redress existing structural inequities in opportunities and financial resources.^2,3^ National and local health equity efforts in health measurement aim to rectify inequities by improving the collection, reporting, and stratification of race, ethnicity, and sexual orientation and gender identity data to identify subpopulations in need of additional or tailored resources.^4–7^ However, immigrants have largely been excluded from these updated categorizations. For example, the Office of Management and Budget recently updated racial, ethnic, and national origin categories to improve data collection and reporting across federal agencies, yet did not update terminology related to immigration status.^8^ Moreover, the Centers for Medicare & Medicaid Services (CMS) released a technical resource on suggested health equity standards for data elements that also does not mention immigrant status or immigrant health as part of the guidance document.^9^ Missing or inaccurate data related to immigrant populations hinders the adequate assessment of the state of access, quality, and coverage in this population. Using humanizing language regarding immigration status is one step to improve health measurement and advance health equity related to immigrant populations.

Societal-level factors, including hostile and dehumanizing anti-immigrant rhetoric (particularly in legal and policy materials), adversely affect health outcomes among immigrants.^10^ For example, studies show associations between anti-immigrant rhetoric with delayed emergency room utilization and increased mental health burden.^11–13^ However, limited empirical research has examined causal mechanisms between dehumanizing language and health outcomes in immigrant communities. To address this gap, recent scholarship has introduced the concept of migration stigma, a novel public health framework explaining how politics shape health through social processes namely by identifying migrants as a stigmatized group, thereby triggering stereotyping, status loss, and discrimination.^14^ Building on this idea, newer studies have focused on improving conceptual clarity around migration stigma, anti-immigration rhetoric, and policy and legal language in relation to health.^10,14^

Our conceptualization of humanizing language integrates concepts of human dignity and ethical respect for identity integrity. Human dignity is defined as an inherent attribute of all human beings that is not dependent on one’s social status, color, gender, nationality, religion, place of birth, sexual orientation, or physical or mental ability.^15^ Identity integrity suggests that no individual or collective identity group should be subjected to subordinating social conditions or processes as the result of efforts to access humanizing opportunity structures.^16^ All humans, by lieu of existence, reserve the right to access essential resources and be protected from risks such that they can manifest their greatest potential.

Today, immigrants of varying statuses represent over 46 million or 14% of all people residing, living, and working in the United States.^17^ In 2022, people who identified as Latino, Hispanic, or Latinx comprised the largest ethnic group among immigrants.^17^ Dehumanizing language used to characterize immigrant populations represents one aspect of anti-immigrant rhetoric and discrimination that can adversely affect mental health and wellbeing in the United States.^18,19^ Use of dehumanizing terms further excludes immigrants from health equity policy discussions to justify the notion that immigrants do not qualify for health insurance or access to health services. Additionally, this divisive language and framing reinforce negative public attitudes toward immigrant populations, creating a feedback loop that increases psychological dehumanization, which, in turn, perpetuates structural racism and ethnic discrimination.^20^

In this perspective, we conceptualize the terms “alien” and “illegal alien” as forms of discriminatory or dehumanizing language toward any immigrant population. Research rooted in Integrated Threat Theory has demonstrated that dehumanizing terminology, specifically “illegal aliens,” invokes heightened perceptions of both symbolic and realistic threats compared to the more neutral term of “undocumented workers.”^21^ These perceived threats drive prejudice by reinforcing negative stereotypes, intensifying discriminatory attitudes, and fostering exclusion and marginalization. As a result, dehumanizing language promotes a social and political climate that legitimizes hostility and discrimination toward immigrants, and discrimination has well-documented detrimental effects on health outcomes and mediators.^14,22–24^

In prior work, we defined health equity as “the just opportunity to attain optimal health and well-being by addressing imbalanced processes, policies, and power that create avoidable differences in health outcomes and allocation of resources.”^25^ Here, we describe specific terms of dehumanizing language associated with immigrant communities in the United States and offer more humanizing alternatives for health data collection and reporting. For example, using the more descriptive and humanizing term “residents without accepted identification” is one among several preferred alternatives to the dehumanizing term “illegal alien.” The policy choice to use “illegal alien” creates avoidable differences in health outcomes by increasing social risk and impeding access to health-promoting resources and services. We recommend adopting equity-centered policy to promote the use of humanizing language when referring to immigrant populations in health equity data collection and reporting.

## Language as a Form of Structural Exclusion in Health Data

An often-overlooked form of exclusion in health care is the use of dehumanizing language in health data, including in publicly available data files and health insurance enrollment resources. For example, North Carolina’s Family and Children Medicaid Manual and Texas’s Health and Human Services Handbooks repeatedly refer to noncitizen individuals as “aliens” and “illegal aliens.”^26–28^ Similarly, Washington State’s Health Care Authority uses the term ‘Alien Medical Programs’ for health care services provided to residents ineligible for public insurance programs, and both Georgia’s Medicaid and CMS’s Medicare Part A and B enrollment resources also reference “aliens.”^29–31^ This terminology may partially stem from its precedent in federal rulings, such as *Arizona v. United States* and *De Canas v. Bica*, which explicitly employ the term “illegal aliens,” as well as federal legislation such as the Personal Responsibility and Work Opportunity Reconciliation Act of 1996 and the Immigration and Nationality Act of 1952, where “alien” is used synonymously with noncitizen.^32–35^ However, using such language in health settings is both inappropriate and unjust. It violates the medical ethics principles of justice, which mandates the fair and equitable treatment of all individuals, and nonmaleficence, which obligates health professionals to avoid causing harm to patients, as well as undermines core values of the public health code of ethics.^36,37^

Immigration status information can show up in health data systems in electronic health records or clinical notes (e.g., in social determinants of health screening questions) and is increasingly being documented in states such as Texas and Florida as new laws are requiring health systems to ask about citizen status.^38^ Immigration status also may appear in public health surveys and surveillance systems (e.g., questions about citizenship, country of birth, and length of residence), research studies on health disparities, policy, and administrative data sets (e.g., eligibility for public health programs and benefits or data linkage between public health agencies and immigration enforcement agencies). Without accurate equity-informed data collection and reporting practices in these contexts, researchers and providers may act on biases and stereotypes that jeopardize positive interactions with health systems and worsen health equity for Hispanic, Latino, and Latinx-identifying populations.^39^

Selecting dehumanizing terms such as “illegal aliens” in official health data and reporting perpetuates harmful rhetoric and reinforces exclusionary attitudes, contributing to a social and political climate that legitimizes hostility and discrimination toward immigrant communities. This rhetoric, which increases social vulnerability and acculturative stress, has been associated with increased behavioral, mental, and physical health burdens and delayed utilization of critical health services among Latinx immigrants.^40–46^ By embedding such language in health data, government agencies risk perpetuating structural racism and creating an environment in which denying care based on immigration status is normalized, undermining broader efforts to achieve health equity.

During the last few years, there has been a proliferation of needed guides that offer tools to redress structural racism and encourage a shift toward antiracist language in public communications.^47,48^ Health equity language guides have noted terms to use and avoid in health communications and analyses, including terms that implicitly or explicitly refer to immigrant communities.^49^ For example, terms such as “hard-to-reach” have been criticized for placing the onus of blame for poor health outcomes on individuals rather than on the systems around them that create inaccessible, unaffordable, and misaligned health resources in relation to community priorities. A suggested alternative is socially marginalized or underrepresented populations/demographics. Use of the term marginalized reflects institutionalized social processes that actively identify and relegate to the fringes select populations groups. Use of the term marginalized also implicates identity-based structural inequalities as the fundamental causal mechanisms reliably relegating select population groups to vulnerable positions. Notably, the Associated Press and several news organizations removed the term “illegal alien” from use in writing and reporting over a decade ago.^50,51^ Many federal agencies and professional organizations have also advocated for using the term “undocumented” as a preferred alternative term to “illegal alien.”^52–54^ While “undocumented” is an improvement to the term “illegal alien,” it conceals underlying social processes that lead to increased social susceptibility and marginalization.

Language, through the explicit selection or omission of terminology, can act as an oppressive mechanism that bolsters structural violence and structural racism, two concepts with well-established historical and contemporary evidence in the United States.^55^ In Table 1, we offer illustrative examples of dehumanizing terminology often used to refer to immigrant populations, reasons why these terms are problematic and humanizing alternatives. Efforts to advance language equity in health have focused on linguistic inclusivity by providing written and verbal communication in varying preferred languages.^56^ For example, the term “Limited English Proficiency” has become a term to refer to populations who may not speak English. However, experts have raised concerns that using this term creates a binary construct—grouping individuals into whether or not they consider themselves “English proficient” regardless of the setting in which English is needed (e.g., retail vs. medical setting), highest level of education, and number of languages spoken.^57^ Furthermore, this term reinforces a narrative of homogeneity among heterogenous multicultural and multilingual immigrant communities.^56^ Similarly, the use of the term “illegal alien” in data collection and reporting harms patients who live, work, and reside in communities across the United States and territories by explicitly placing value on and dignifying some lives over others. This dehumanizing term perpetuates a misconception that immigrants do not contribute to society through labor or taxes when, in fact, immigrants regardless of immigration status pay local, state, or federal taxes funding social services. Significantly, immigrants contribute to society in many other critical ways, as evidenced by the high number of immigrants identified as crucial essential workers during the COVID-19 pandemic.^58^

**Table 1.** Current Terms and Illustrative Humanizing Alternatives

Terminology	Reason this term is problematic	Alternatives
Illegal Alien or Alien	Legality is tied to immigration regulations and frameworks that evolve. A person’s legal status is not an inherent individual characteristic that the use of the term “illegal” conveys.	Residents/People without accepted forms of identification
Undocumented	While an improvement compared to the term “illegal,” “undocumented” implies that the lack of documentation status equates to a community attribute without adequate interrogation of what mechanisms place a person at greater risk.	
Limited English Proficiency	Creates a binary measure that stigmatizes individuals or communities with different proficiency of a dominant language. Does not account for proficiency levels that can be different for every day conversation compared to conversations in a medical setting that may require translation.	Preferred language choice of (select language)
Hard to Reach	Implies vulnerability is a condition inherent and internal to the group. Conceal social, economic, and political processes that produce and maintain vulnerability. Places onus of addressing deficiencies in health access, affordability, and availability on communities rather than on health decision makers in policy, research, and clinical practice.	Hardly reached Socially marginalized or excluded populations

## Implicit Message of Health Equity for Some

Immigrant communities, the majority of whom originate from countries in Latin America and Asia, also often experience systemic exclusion, which we previously defined as “occur[ing] when communities experience continuous and compounded legal, regulatory, and political injustices that hinder a population’s ability to achieve health and wellbeing.”^59,60^ The inclusion of the term “illegal alien” in health data collection and reporting places another layer of stigma on the most marginalized immigrants, many of whom experience social exclusion, despite their critical contributions to the social fabric and economy of society.

The term ‘illegal alien’ is used to refer to all immigrants who lack documentation in the United States, regardless of race, ethnicity, or nationality. Nevertheless, multiple studies demonstrate that political and media messaging disproportionately frames ‘illegal immigrants’ as Hispanic, Latino or Latinx, even irrespective of documentation or citizenship status.^61–64^ This phenomenon is referred to as racialized legal status.^65^ Even if unintentional, the consequence is that people in one ethnic group experiencing compounded marginalization across political, legal, economic, and health systems are further discriminated from receiving health services due to a value judgement placed on their being. These consequences are heightened given that Hispanic, Latino or Latinx populations, which the US Census defines as individuals from all Spanish-speaking countries in the Latin America and Caribbean region, bear the brunt of the immigration enforcement system in the United States today.^61,66^ As others have noted, the inclusion of the term “illegal alien” produces an othering effect and creates a social hierarchy of two groups of people: those that are humanely dignified beneficiaries of health equity efforts and those that are not.^67,68^ Therefore, integrating language and data equity principles in data collection and reporting efforts is an essential policy step for improving Latinx health outcomes.

## Ethical Considerations in Data Collection

It is essential that clinicians, researchers, and health policymakers act with caution when collecting data of individuals seeking care in their health system. For immigrants, engaging with authorities poses inherent threats, including increased surveillance, policing, and threat of deportation, to people or family members of individuals who lack accepted forms of documentation. The changing political climate at the federal and state level heightens risks immigrant communities face in accessing health services.^69,70^ However, all people in the United States, regardless of legal status, utilize health services at some point in time. Therefore, it is important to use descriptive or identifying language and other social processes that honor these principles of human dignity and identity integrity. Terms or systems that do not do so are defined as dehumanizing.

Before collecting data on documentation status, it is critical to rigorously evaluate its necessity, purpose, and the ethical implications of doing so. Public health leaders, researchers, and policymakers must carefully weigh the potential benefits of such data, which can improve our understanding of health disparities, against the significant risks it poses to populations experiencing increased vulnerability including stigmatization, denial of services, or even deportation.^71^ Ethical considerations, grounded in public health principles, require a thorough assessment of whether these data are truly essential or if the same objectives can be achieved through alternative, less sensitive measures. Federal privacy laws, including Health Insurance Portability and Accountability Act, provide some protections against disclosure, but exceptions for law enforcement requests or variations in state law may still expose patients to harm.^72,73^ If collecting such sensitive data is deemed necessary, it must be accompanied by clear protocols for data protection (e.g., encryption and strict deidentification protocols), informed consent procedures, and safeguards against misuse, with particular attention to preserving the trust and privacy protections previously afforded by policies such as the designation of health care facilities as “Protected Areas” under Department of Homeland Security guidelines. For example, researchers at the Hispanic Health Disparities Research Center at the University of Texas, El Paso, employed stringent measures by destroying identifiers once data collection was complete, ensuring no records could expose participants to harm.^74^ Investigators may also obtain Certificates of Confidentiality from the National Institutes of Health, which enable them to refuse compliance with subpoenas or court orders seeking participant identifiers, providing an additional layer of legal protection.^75^

## Policy Recommendations to Encourage Use of Humanizing Language to Improve Health Equity

**FIG. 1.**
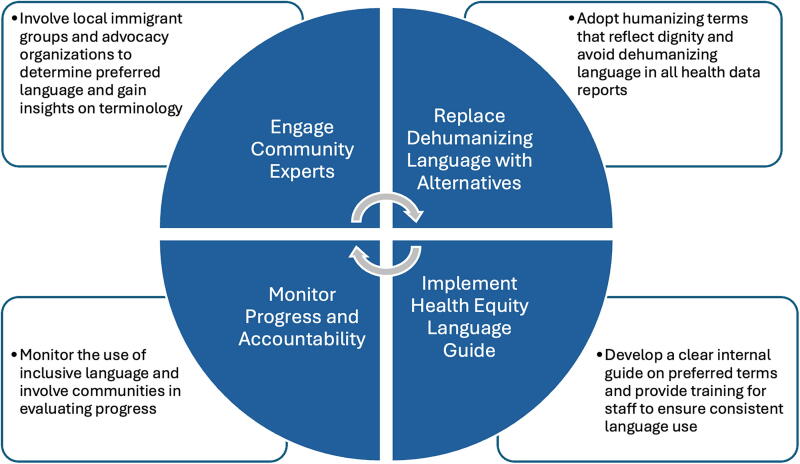
Steps for Implementing Humanizing Language in Health. Equity Data Collection.

The long-standing use of “illegal aliens” in health data collection and reporting systems hinders health equity goals that policy makers aim to accomplish and can contribute to migration stigma. Figure 1 illustrates the types of actions different health actors can take to mitigate this gap in health equity. These actions are not undertaken once but repeated, refined, and reinformed by ongoing community engagement, ensuring that health equity language practices evolve and improve over time.

In addition to upholding the ethical considerations previously discussed, engaging affected communities is essential to understanding preferences and concerns, including whether documentation status should be collected at all. If communities’ express opposition, their input should guide decision-making. Ultimately, any decision to collect these data must be grounded in a well-justified public health purpose, uphold robust privacy safeguards, and prioritize the autonomy, rights, and safety of immigrant communities.

If it is determined that collecting data on documentation status is necessary, the following steps should guide efforts to ensure ethical, inclusive, and community-centered practices.

First, policy makers at local or state departments of health and human services or administrators at health systems can adopt alternative terminology that better reflect the contributions, value, and social fabric that all community members provide to society. Terms such as “people without legal status,” “people—undocumented,” or “people lacking documentation” are improvements compared to “illegal alien,” yet still mask the effects of policy choices that lead to the systemic exclusion of marginalized populations. A term such as “people without accepted or qualifying documentation” more accurately reflects the reality that many immigrants have some forms of documentation but lack those documents that provide access to health services in the United States (e.g., a state issued ID that is required for pharmacy- and hospital-based services). Notably, after receiving feedback about the inclusion of the terms “Aliens-illegal” and “Aliens” as categories in the March 2024 Medicaid enrollment spreadsheet, North Carolina Department of Health and Human Services (NCDHHS) removed these terms from the listed categories.

Second, when making determinations about appropriate terms to use to describe an attribute or identity, it is important that health providers, researchers, and policy makers center preferences of the identified communities in the generation of those “labels.” For example, if local departments of health and social service organizations are determining how to track how “people lacking documentation” are faring on a specific health outcome that has been prioritized, it is important to garner input from that specific community on their preferred terms. For example, researchers and local public health leaders will benefit from collaborating with local coalitions to identify terms that humanize immigrant populations within their states. Some communities may prefer to not include immigration status while communities may consider it a necessity based on population demographics. Local leaders can implement community-based or community-engaged strategies to achieve this goal. Practically, this collaboration could involve developing a community advisory council on health equity language whose members can serve as a collective group to discuss preferred wording or terms. Importantly, no one person, representative, or organization can offer a definitive opinion of all community members, which is why it is critical to engage multiple people or organizations. In the same way that policy makers engage experts in the development of policies or interventions, engaging people who are most affected by the discriminatory ramifications of dehumanizing language will strengthen credibility and offer opportunities to build trust.

Third, monitoring and evaluating progress in health equity language can help ensure the inclusion of all marginalized populations in state health equity initiatives. Data equity initiatives are crucial to ensure populations are not overlooked in public reporting or analyses that are used to inform policy decision-making. Public sector leaders in local, state, and federal governments can adopt a health equity language guide to ensure principles of health equity are integrated into data collection and reporting efforts. To evaluate uptake of these strategies, leaders can report which guide is adopted and track the percentage of public-facing and internal documents reviewed and edited to use such language. The establishment of an accountability mechanism which includes the engagement of community members would demonstrate commitment to humanizing language, allow for tracking of progress, and provide a method of comparison across states and institutions, which would encourage other health leaders to follow suit. Importantly, the insights gained through monitoring and evaluation should guide a return to the community engagement phase, fostering ongoing input from community experts. This ongoing feedback loop allows for the continuous refinement of language and updating of policies, ensuring that efforts to promote health equity remain dynamic and responsive rather than static or one-time interventions.

In the future, national policy makers can establish national data standards across federal agencies to eliminate the use of “illegal alien” from health data collection and reporting. Such national efforts are crucial in reducing state-level differences of continued dehumanization that would occur if the term is not removed from all health agency data collection and reporting guidance.

Advancing health equity requires redressing long-standing and established social, economic, legal, and political determinants of health that create unequal opportunities for some members in our society. Health data is a powerful tool that can help remediate gaps in care, funding, and decision-making, but requires data labels that humanize all populations living, working, and residing across the United States and territories. Language specificity can amplify or hinder how health data can enable improvements in population health for all populations, including those that experience the greatest marginalization. Unfortunately, biased and discriminatory language related to immigration status disproportionately impacts health outcomes of Latinx communities due to the racialization of legal status and subsequent increased use of the term to refer to and describe this ethnic group. Nevertheless, while we have centered our research and advocacy efforts on people of all races who identify as Hispanic, Latino/a or Latinx/e, immigrants from other minoritized racial and ethnic backgrounds experience similar exclusionary and harmful effects of use of dehumanizing terminology. Removing dehumanizing terms such as “illegal alien” from health data collection and reporting efforts will have a positive ripple effect on health outcomes of all immigrant communities and their families.

## Abbreviations Used

CMS: Centers for Medicare & Medicaid Services
NCDHHS: North Carolina Department of Health and Human Services
